# Development of real time PCR for detection and quantitation of Dengue Viruses

**DOI:** 10.1186/1743-422X-6-10

**Published:** 2009-01-23

**Authors:** KR Gurukumar, D Priyadarshini, JA Patil, A Bhagat, A Singh, PS Shah, D Cecilia

**Affiliations:** 1 National Institute of Virology, 20A Dr. Ambedkar Road, Pune 411001, India

## Abstract

**Background:**

Dengue virus (DENV), a mosquito borne flavivirus is an important pathogen causing more than 50 million infections every year around the world. Dengue diagnosis depends on serology, which is not useful in the early phase of the disease and virus isolation, which is laborious and time consuming. There is need for a rapid, sensitive and high throughput method for detection of DENV in the early stages of the disease. Several real-time PCR assays have been described for dengue viruses, but there is scope for improvement. The new generation TaqMan Minor Groove Binding (MGB) probe approach was used to develop an improved real time RT-PCR (qRT-PCR) for DENV in this study.

**Results:**

The 3'UTR of thirteen Indian strains of DENV was sequenced and aligned with 41 representative sequences from GenBank. A region conserved in all four serotypes was used to target primers and probes for the qRT-PCR. A single MGB probe and a single primer pair for all the four serotypes of DENV were designed. The sensitivity of the two step qRT-PCR assay was10 copies of RNA molecules per reaction. The specificity and sensitivity of the assay was 100% when tested with a panel of 39 known positive and negative samples. Viral RNA could be detected and quantitated in infected mouse brain, cell cultures, mosquitoes and clinical samples. Viral RNA could be detected in patients even after seroconversion till 10 days post onset of infection. There was no signal with Japanese Encephalitis (JE), West Nile (WN), Chikungunya (CHK) viruses or with Leptospira, *Plasmodium vivax*, *Plasmodium falciparum *and Rickettsia positive clinical samples.

**Conclusion:**

We have developed a highly sensitive and specific qRT-PCR for detection and quantitation of dengue viruses. The assay will be a useful tool for differential diagnosis of dengue fever in a situation where a number of other clinically indistinguishable infectious diseases like malaria, Chikungunya, rickettsia and leptospira occur. The ability of the assay to detect DENV-2 in inoculated mosquitoes makes it a potential tool for detecting DENV in field-caught mosquitoes.

## Background

Dengue virus (DENV) is a mosquito borne flavivirus with four serotypes DENV-1 to 4. The global prevalence of dengue has grown dramatically in the recent decades. The disease is now endemic in more than 100 countries around the globe and it is estimated that DENV causes 50 to 100 million cases of acute febrile disease every year [1]. Since 1945, outbreaks of dengue caused by all 4 serotypes have been reported regularly from different regions of India [2].

Dengue is diagnosed by either detecting virus or antibody to the virus in blood. Isolation of virus in cell culture or in infant mouse brain remains the gold standard for diagnosis, but it takes more than a week for the test to be completed making it impractical in most situations. Detection of anti-dengue IgM and IgG in the serum by ELISA is the most commonly used criteria for presumptive diagnosis of DENV infections. These serological methods are unable to detect the infection during the early phase of the disease. Thus there is a need for rapid and sensitive methods for detection of DENV early in the course of infection for better patient management. Several PCR based methods for detecting DENV nucleic acid in the serum have been reported, the most widely used test is the nested RT-PCR developed by Lanciotti et al., [3] and a modification of the same method to single tube format by Harris et al., [4]. More recently real-time PCR based methods have been reported for detection and serotyping of DENV which use fluorescent based reporter chemistries [5-8]. Real-time PCR has many advantages over conventional RT-PCR, in that it is more sensitive, can be automated thereby enabling high throughput screening, and the hands on time including sample handling is less than four hours. The real-time PCR is also used to quantitate the viral load in blood samples, making it a useful tool to investigate the role of viremia in pathogenesis of dengue. Another important aspect of dengue disease is the surveillance of vector population and detection of DENV in field caught mosquitoes. Real-time PCR because of its high sensitivity could be useful in such surveillance and provide early warning of a possible outbreak of the disease. Recent reports on DENV group specific real-time PCR used SYBR green based method [9,10] where as an earlier report based on TaqMan probes used multiple probe primer sets for detection of all four serotypes of DENV [11]. In the present study, we describe the development of a DENV-specific TaqMan based real-time PCR for detection and quantitation of all four serotypes using a single probe primer set targeted against the 3'UTR of DENV.

## Methods

### Viruses

Sixteen strains of dengue viruses, including five strains of DENV-1, four strains of DENV-2, three strains of DENV-3, four strains of DENV-4 and one strain each of JE, WN and CHK viruses were obtained from the virus repository of National Institute of Virology, Pune, India, (NIV) as infectious mouse brain stocks (Table 1). DENV stocks were used directly for sequencing and for evaluation of sensitivity of the real-time PCR. JE, WN and CHK viruses were used to evaluate the specificity of the assay.

**Table 1:** Viruses used in the study

Serotype	Strain	Year and location
DENV-1	623993*	1962, Vellore, India
	631287*	1963, Vellore, India
	631289*	1963, Vellore, India
	055290*	2005, Pune, India
	16007^@^	Thailand

DENV-2	P23085*	1960, Vellore, India
	803347*^@^	1980, Kolkata, India
	939548*	1993, Pune, India
	057561*	2005, Pune, India

DENV-3	633798 (H87)	Rockefeller lab, USA
	059827*^@^	2005, Kolkata, India
	059826*	2005, Kolkata, India

DENV-4	611318*	1961, Vellore, India
	624000*	1962, Vellore, India
	631306*	1963, Vellore, India
	642069^@^	1964, Vellore, India

JEV	P20778	1958, Vellore, India

WNV	E101	1950, Cairo, Egypt

CHK	62736	2006, Andhra Pradesh, India

*Strains used to sequence 3' UTR for designing primers and probe in this study.

^@^Strains used to make tissue culture stocks.

### Infection and maintenance of cell cultures

Vero cells were maintained in MEM supplemented with 10% FCS. One strain of each serotype was amplified in Vero cells to make virus stocks for determining standard curves. Cell cultures were infected at 0.1 multiplicity of infection (MOI) with DENV-1 (16007), DENV-2 (803347), DENV-3 (059826) or DENV-4 (642069). The virus was harvested after 4 to 5 days post infection after appearance of cytopathic effect. The infected cell cultures were subjected to a single freeze thaw. The cell lysates were clarified by centrifugation at 2000 rpm and stored in suitable aliquots at -80°C until used.

### Infection of mosquitoes

Female *Aedes aegypti *mosquitoes were inoculated with 10^3 ^PFU/mL of DENV-2 (803347) by intrathoracic route. Infected mosquitoes were kept for 14 days at 28°C and 80–90% humidity. Surviving mosquitoes were frozen at -80°C. The mosquito heads were used for Immuno Fluorescence Assay (IFA) as described earlier [12] and the bodies were used for RNA extraction. A total of 34 mosquitoes including 10 control mosquitoes and 24 mosquitoes inoculated with DENV-2, were tested by IFA and qRT-PCR.

### Clinical samples

Three hundred and eight blood samples were used to evaluate the usefulness of the qRT-PCR assay for its diagnostic potential. The blood samples were obtained within 10 days post onset of symptoms. The sera were separated, aliquoted and stored at -80°C. All samples were tested for presence of DENV RNA by nested RT-PCR as described earlier [3] and dengue specific IgM antibodies using the NIV MAC-ELISA kit [13]. Serum samples positive for other febrile illnesses i.e. Leptospira (n = 2), Rickettsia (n = 1), and Malaria (n = 2) were provided by Dr. RR. Gadia of King Edward Memorial Hospital, Pune India. Paired serum samples (n = 4), which were confirmed to be dengue by a four fold rise in HI titre [14] were also included to analyse the sensitivity of the qRT-PCR. Serum samples from acute cases of CHK and JE were not available therefore sera from healthy individuals were spiked with 10^5 ^PFU of CHKV or 10^3 ^PFU of JEV and used to evaluate the specificity of the qRT-PCR.

### Primer and probe design

The 3'UTR of four strains of DENV-1 (nt 10230–10700), four strains of DENV-2 (nt 10301–10708), two strains of DENV-3 (nt 10243–10689), and three strains of DENV-4 (nt 10315–10635) isolated in India (Table 1) were sequenced using the big dye terminator kit (Applied Biosystems, Foster city, CA, USA). These 13 sequences were aligned with sequences of 11 DENV-1 strains, 11 DENV-2 strains, 13 DENV-3 strains and 6 DENV-4 strains from GenBank using the Clustal W programme. A stretch of nucleotides conserved in the four serotypes was identified and the primers and probe sequences were designed using the Primer Express software.

### Generation of RNA standard for the qRT-PCR

The target region from the 3'UTR was amplified from DENV-3 (633798) and cloned in to the TEasy cloning vector (Promega Corporation, Madison, WI, USA). The presence and orientation of the insert DNA was confirmed by sequencing. The plasmid was linearized by digestion with *ApaI *and the target sequence was amplified using the *in vitro *RNA transcription kit (Roche Diagnostics, IN, USA). The transcribed RNA was treated with DNAse to digest the plasmid and purified using the QIAamp RNA purification kit (Qiagen Sciences, Valencia, CA, USA). The RNA was quantified by spectrophotometry. The copy numbers of the RNA was calculated based on the concentration and its molecular weight and 10 fold serial dilutions of this RNA from 10^2 ^to 10^8 ^copies per reaction was used as standard in all qRT-PCRs.

### qRT-PCR

RNA from 140 μl of 10% mouse brain suspension, cell culture lysate or human serum samples was extracted using the QIAamp viral RNA extraction kit (Qiagen Sciences, Valencia, CA, USA) as per the manufacturer's protocol. The RNA was eluted in 60 μl of elution buffer and stored at -80°C. 10 μL of extracted RNA was used in all qRT-PCR. In case of mosquitoes, the entire mosquito was homogenized and the RNA was extracted from the homogenate using TRIZOL reagent (Invitrogen, Carlsbad, CA, USA) as per the manufacturer's protocol. The RNA was resuspended in 20 μl of RNAse free distilled water and stored at -80°C. 0.5 μL of this RNA was used in the qRT-PCR. The transcribed or viral RNA was reverse transcribed using the reverse primer with AMV reverse transcriptase (Promega Corporation, Madison, WI, USA). The reverse transcription reaction was carried out at 42°C for 1 h. The cDNA thus obtained was used as the template in the qPCR. The TaqMan universal PCR master mix (Applied Biosystems, Foster city, USA) was used in all qPCRs. Each reaction had 200 nM of forward primer, 250 nM of probe and 300 nM of reverse primer in a 25 μL final reaction volume. The PCR mixtures were pre incubated at 50°C for 2 min followed by denaturation at 95°C for 10 min and 45 cycles of 95°C for 15 sec and 60°C for 1 min using the Applied Biosystems 7500 real-time PCR system. The real-time data was analyzed using the SDS software provided by Applied Biosystems.

## Results

### Design and evaluation of primers and probes

**Figure 1:**
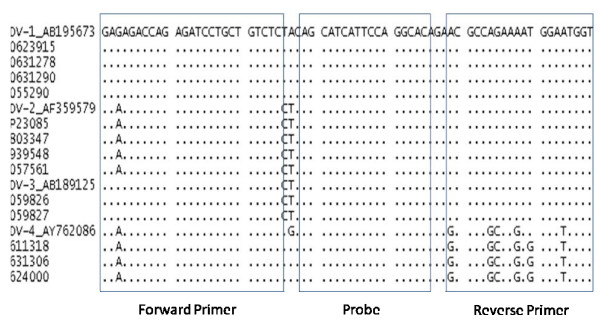
**Clustal W alignment of the DENV 3'UTR region sequenced by us and representative sequence of each serotype of DENV downloaded from the gene bank**.

Alignment of the 3'UTR sequences of 13 Indian strains of DENV and the 41 sequences from GenBank revealed that the region was highly conserved within each of the four serotypes but variable between serotypes. A single stretch of 100 nucleotides was found to be highly conserved among the four serotypes of DENV, except for a six base mismatch in DENV-4. The 100 bp region from nt 10628 to nt 10728 was used to design the primers and probe (Fig. Fig. 1). A generic reverse primer (nt 10708-10682), a forward primer (nt 10635–10658) and the probe (nt 10663–10679) were synthesized (Table 2). The probe was labelled with FAM at the 5'end and a minor groove binder (MGB) and a non fluorescent quencher at the 3'end.

**Table 2:** Nucleotide sequence of primers and probe used in the qRT-PCR assay

	Sequence	Nucleotide position
Forward Primer	5'-GARAGACCAGAGATCCTGCTGTCT-3'	10635–10658
Reverse Primer	5'-ACCATTCCATTTTCTGGCGTT-3'	10708-10682
TaqMan MGB Probe	5'-AGCATCATTCCAGGCAC-3'	10663–10679

**Figure 2:**
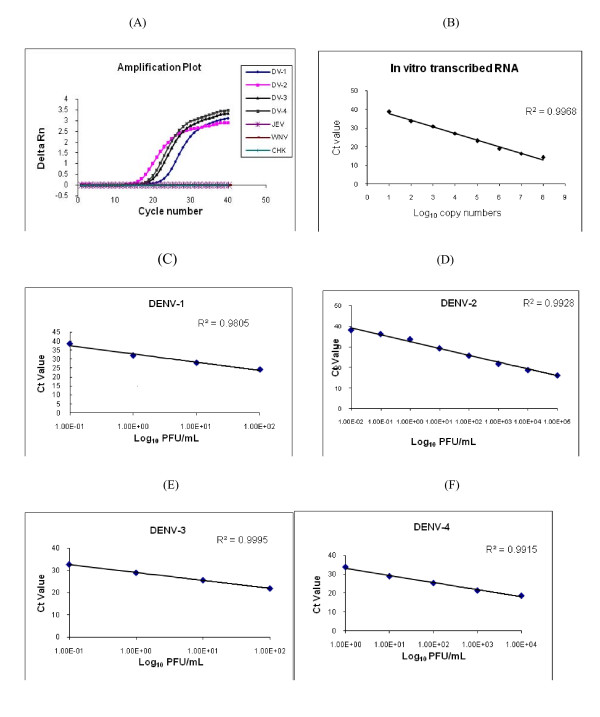
**Specificity and sensitivity of the qRT-PCR assay**. (A) Amplification plots obtained for DENV-1 to 4 viruses, (B) standard curve of the in vitro transcribed RNA and (C-F) standard curves for the four DENV serotypes.

The specificity of primers and probe were tested against the four serotypes of DENV, JE, WN, and CHIK viruses. Fig. 2A shows the amplification plot generated for the different viruses. Amplification of the four serotypes of DENV was observed from 15 cycles onwards as indicated by an increase in the fluorescence intensity. The fluorescence intensity values for the other viruses remained at the base line similar to the 'no template controls' indicating that the test successfully detected all the four DENV serotypes but did not show any amplification of JE, WN, and CHK viruses.

### Sensitivity and Specificity of the qRT-PCR assay

RNA extracted from fourteen DENV strains (Table 1) obtained from the Institute's virus repository and fifteen serum samples which were confirmed to be positive for DENV by virus isolation (n = 11) or by a four fold rise in HI titre in paired serum samples (n = 4) were used to evaluate the sensitivity of the qRT-PCR assay. The assay could detect DENV RNA in all the DENV positive samples indicating that the test was 100% sensitive in detecting DENV RNA [(number of positive specimens/(number of positive specimens + number of false negative specimens)].

The specificity of the assay was evaluated using RNA extracted from virus stocks of two related Flaviviruses (JE and WN), and CHK virus, which causes a dengue like disease and is transmitted by the same vector. In addition RNA extracted from serum samples spiked with JE or CHK viruses and from sera of confirmed leptospira (n = 2), rickettsia (n = 1) and malaria (n = 2) cases were also used for determining the specificity of the assay. None of the 10 samples tested by the qRT-PCR assay showed positive amplification, suggesting that the test was 100% specific for the detection of DENV [number of negative specimens/(number of negative specimens + number of false positive specimens)].

The detection limit of the assay was evaluated by both RNA copy numbers and by PFU. Ten fold serial dilutions of the *in vitro *transcribed RNA was used to determine the sensitivity of the assay. The minimum that could be detected was 10 copies of RNA molecules per reaction as indicated in the standard curve (Fig. 2B). For quantitation by PFU, ten fold dilution of RNA extracted from virus stocks of all four serotypes of DENV with known PFU titers were used. The PFU titers of the stock viruses ranged from 1.3 × 10^3^to 1 × 10^6^/ml (Table 3). The detection limit of the assay varied for the four serotypes of DENV, the sensitivity of detection was highest for DENV-2 and lowest for DENV-4 (Fig. Fig. 2C–F, Table 3).

**Table 3:** Detection limit of the qRT-PCR assay for the four serotypes of DENV

Serotype(Strain)	Titer PFU/mL	Detection limit (PFU)	Correlation coefficient
DENV-1 (16007)	1.3 × 10^3^	0.1	0.98
DENV-2 (803347)	4.2 × 10^6^	0.001	0.99
DENV-3 (059826)	1.4 × 10^4^	0.1	0.99
DENV-4 (642069)	3.5810^5^	1	0.99

### Detection and Quantitation of DENV in human samples

**Figure 3:**
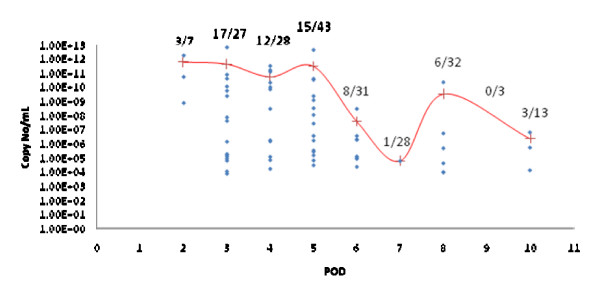
**Viral load quantitated by qRT-PCR**. Viral RNA copies/ml in sera collected from dengue patients on different post onset days. The mean of the observations obtained for each POD is depicted as (+).

For evaluating the usefulness of the qRT-PCR for detecting DENV in clinical samples, 308 sera from dengue suspected cases were tested by MAC-ELISA, nested RT-PCR and qRT-PCR. Of the total samples tested, 212 (68.8%) were positive for dengue infection by one of the three methods described. Among the 212 dengue positive cases, 65 (30.7%) were positive for viral RNA by qRT-PCR and 36 (16.9%) by nested RT-PCR. All nested RT-PCR positive samples were also positive by qRT-PCR. Of the 186 IgM positive samples 33 (17.7%) were positive for DENV RNA by qRT-PCR whereas only 15 (8.1%) were positive by nested RT-PCR. Therefore qRT-PCR was found to be highly sensitive (p < 0.01, McNemar's test) in detecting DENV when compared to RT-PCR. The minimum amount of RNA detected in these samples was 1.04 × 10^4 ^copies/mL and the maximum amount detected was 6.9 × 10^12 ^copies/mL of serum. The mean viremia was higher during the initial days of infection and decreased during later stages of infection (Fig. 3). The number of samples positive by qRT-PCR during 2–5 days post onset of fever (47/105) was highly significant (p < 0.01, Chi-square test with Yates correction) when compared with samples collected from 6–10 days post onset of fever (18/107). The inverse was true for IgM, where in the detection of IgM was significantly higher (p < 0.01, Chi-square test with Yates correction) in the samples collected from 6–10 days post onset of fever (104/107) when compared to the 2–5 days post onset of fever (82/105).

## Discussion

Several real-time PCR based methods for detection of DENV have been reported in the last decade. These assays have targeted the 3'UTR [9,15], NS5 [11,16], core [9] and the envelope [17] gene sequences. Majority of the recent reports describe development of the serotype specific real-time PCR for dengue using TaqMan probes [8,18] or FRET probes [6]. Though these methods are useful for serotyping of DENV they may not be cost-effective for routine diagnosis as only a small percentage of samples are positive for DENV RNA during the non endemic season and during the active transmission season only about 50% of samples may be positive for DENV RNA [6]. A group specific PCR would be a useful tool for initial screening of the samples and only those samples positive for DENV can then be subjected to serotyping. Lai et al., [6] initially screened samples by a SYBR green based group specific real-time PCR and then serotyped the positive samples by a duplex or a fourplex TaqMan based assay, thereby reducing the operation cost on diagnosis by half. The SYBR green-based detection systems have the major disadvantage of false positives due to dye binding to primer dimers or to DNA amplified non-specifically. Melt curve analyses is often used to confirm the fidelity of the reaction. Other group specific real time PCR assays reported are also SYBR green based [9,10,19]. The first DENV group specific assay using TaqMan probes was described by Callahan et al., [11]. In the assay the authors have used multiple probe primer sets for establishing a group specific RT-PCR assay. This could have been because of lack of a suitable stretch of conserved nucleotides for designing the conventional TaqMan probes, earlier observed by Laue et al., [16]. The new generation MGB probes can be much shorter in length because of the Minor groove binder tagged to the probe, which increases the thermal stability of the probes. We used the TaqMan MGB probe to develop the group specific assay in this report which made it possible to use the short conserved region in the 3'UTR of the dengue genome to design the assay using a single probe and primer pair combination. The fluorescent probes in TaqMan assay are known to be target specific and sensitive to mismatches [20]. To avoid false negative results due to mismatches in the probe binding sites, the 3'UTR region of thirteen Indian strains, representing all four serotypes, isolated from different regions in India were sequenced. These sequences were aligned with representative sequences of all four serotypes from the GenBank and a conserved region of 100 nucleotides was used to design the primers and probe.

All four serotypes of DENV from infected mouse brain or cell cultures or mosquitoes or clinical samples could be detected by the qRT-PCR. No amplification with the related Flaviviruses or with samples from other febrile illness was observed. The sensitivity of the qRT-PCR in terms of PFU was highest for DENV-2 at 0.001 PFU followed by DENV-1 and DENV-3 at 0.01 PFU and DENV-4 had the lowest sensitivity of 1 PFU. The difference in sensitivity could have been due to a difference in the proportion of non-infectious RNA transcripts to infectious particles. The mismatches between the DENV-4 virus sequence and the reverse primer could have contributed to the lowest sensitivity of 1 PFU. An alternate reverse primer with DENV-4 sequence can be included to bring the sensitivities at par. The qRT-PCR assay was able to detect viral RNA in a significantly larger number of clinical samples (30.7%) than RT-PCR (16.9%) (p < 0.001, Mc Nemar's test). The qRT-PCR also detected DENV RNA in a larger number of IgM positive samples when compared to the nested RT-PCR. There is increasing recognition of the importance of viral burden in the pathogenesis of DHF and a direct association between viral load and severity of the disease has been reported [21-23] In earlier studies on viral load the method used to quantitate the virus in the blood samples were either mosquito infectious dose (MID_50_) or PFU. It has been difficult to compare any two studies as the measures vary depending upon the strain of the virus and the substrate used i.e. mosquito or cell line. None of the group specific real-time PCRs reported [6,9-11,24] developed a quantitative RNA standard. We developed an RNA standard for quantitating DENV RNA in this study. The RNA copy number, which offers minimum variability, was used to quantitate the viral load in human clinical samples. Viremia was found to be higher in the initial days of the illness decreasing gradually during the later stages of the infection. This result is consistent with earlier findings based on mosquito inoculation and serotype specific real-time PCR [15,25].

An important aspect of dengue disease control is vector surveillance. Detection of DENV positive mosquitoes will be useful to monitor the infection rates within vector mosquito population and provide an early warning signal to predict an impending epidemic [26]. Currently IFA, insect bioassay, ELISA and RT-PCR are the methods available for detection of DENV in mosquitoes [27]. The qRT-PCR assay could detect DENV-2 RNA in all the inoculated mosquitoes. The sensitivity of detection was such that a 40^th ^fraction of the infected mosquito body lysate was sufficient to give a positive signal in the qRT-PCR, thus even low levels of viral RNA in infected mosquitoes should be detected with the assay. No false positives were observed with control mosquitoes. However testing of field caught mosquitoes by the qRT-PCR will prove its usefulness for vector surveillance.

## Conclusion

The group specific real-time PCR developed in this study will be a useful tool for differential diagnosis of dengue fever in a situation where a number of other diseases like malaria, Chikungunya, rickettsia and leptospira co-exist and are clinically indistinguishable. In areas where other flaviviruses are circulating, IgM detection is not conclusive because of cross reactivity that exists between DENV, JEV and WNV. The ability of the qRT-PCR assay to detect DENV RNA in seropositive individuals up to 10 days post onset of fever is an advantage in such situations. The ability of the assay to detect DENV-2 in inoculated mosquitoes makes it a potential tool for detecting DENV in field caught mosquitoes.

## Competing interests

The authors declare that they have no competing interests.

## Authors' contributions

GKR carried out the sequencing of 3'UTR of DENV strains, standardised the real-time RT-PCR and drafted the manuscript. PD prepared virus stocks in cell culture and performed plaque forming unit assays. BA did the mosquito inoculation experiments and IFA. PJA did the multiplex PCR for clinical samples and participated in the sequence alignment. SA did the MAC ELISA and HI assay for all clinical samples. PSS was involved in getting the clinical samples and the positive and negative controls. CD was involved in conception of the study, drafting the manuscript and revising it critically. All authors have read and approved the final manuscript.
